# Therapeutic Approaches to Tuberous Sclerosis Complex: From Available Therapies to Promising Drug Targets

**DOI:** 10.3390/biom14091190

**Published:** 2024-09-21

**Authors:** Elena Conte, Brigida Boccanegra, Giorgia Dinoi, Michael Pusch, Annamaria De Luca, Antonella Liantonio, Paola Imbrici

**Affiliations:** 1 Department of Pharmacy—Drug Sciences, University of Bari “Aldo Moro”, 70125 Bari, Italy; elena.conte@uniba.it (E.C.); brigida.boccanegra@uniba.it (B.B.); giorgia.dinoi@uniba.it (G.D.); annamaria.deluca@uniba.it (A.D.L.); antonella.liantonio@uniba.it (A.L.); 2 Institute of Biophysics, National Research Council, 16149 Genova, Italy; michael.pusch@ibf.cnr.it

**Keywords:** tuberous sclerosis complex, mTOR, vigabatrin, everolimus, CLC-5, Kv1.1

## Abstract

Tuberous sclerosis complex (TSC) is a rare multisystem disorder caused by heterozygous loss-of-function pathogenic variants in the tumour suppressor genes TSC1 and TSC2 encoding the tuberin and hamartin proteins, respectively. Both TSC1 and TSC2 inhibit the mammalian target of rapamycin (mTOR) complexes pathway, which is crucial for cell proliferation, growth, and differentiation, and is stimulated by various energy sources and hormonal signaling pathways. Pathogenic variants in TSC1 and TSC2 lead to mTORC1 hyperactivation, producing benign tumours in multiple organs, including the brain and kidneys, and drug-resistant epilepsy, a typical sign of TSC. Brain tumours, sudden unexpected death from epilepsy, and respiratory conditions are the three leading causes of morbidity and mortality. Even though several therapeutic options are available for the treatment of TSC, there is further need for a better understanding of the pathophysiological basis of the neurologic and other manifestations seen in TSC, and for novel therapeutic approaches. This review provides an overview of the main current therapies for TSC and discusses recent studies highlighting the repurposing of approved drugs and the emerging role of novel targets for future drug design.

## 1. TSC Phenotype and mTOR Pathway

Tuberous sclerosis complex (TSC) is a rare neurocutaneous disorder caused by heterozygous loss-of-function pathogenic variants in the tumour suppressor genes TSC1 and TSC2 encoding the tuberin and hamartin proteins, respectively [1]. These proteins constitute, together with TBC1D7 protein, a heteromeric complex critical for the negative regulation of the mammalian target of rapamycin (mTOR), a highly conserved serine/threonine kinase involved in the control of cell metabolism, protein synthesis, growth, differentiation, and migration in response to nutrients and growth factors [2,3]. The incidence of TSC generally falls between 1:6000 and 1:10,000 of live births [4].

The TSC phenotype is characterized by multisystem hamartomas (benign tumours), most commonly involving the, brain, kidneys, lungs, heart, skin, and eyes, and is associated with seizures and intellectual disability [1,5]. Brain involvement in TSC includes epilepsy, cortical tubers, subependymal nodules (SENs), and/or subependymal giant cell astrocytomas (SEGAs). Cortical tubers are clusters of abnormal neurons and glial cells in the cortex, resulting from disrupted cellular differentiation and neuronal migration. They are found in 88% to 100% of patients [6,7], and may be linked to seizures and autism spectrum disorders [8]. SENs are small lesions in the subependymal space of the lateral ventricle, with histopathological features like SEGAs. SENs are asymptomatic nodules that may calcify or gradually grow until, by the second decade of life, they transform into formations ≥15 mm in diameter [3,9]. Compared to SEGAs, SENs are smaller, do not grow, and do not show contrast enhancement with gadolinium. SEGAs are benign, low-grade, non-infiltrative tumours of glioneuronal origin. They are defined as caudothalamic sulcus lesions larger than 1 cm in size, or subependymal lesions in any location that show serial growth on consecutive images, regardless of size. SEGAs are often an important source of TSC-related morbidity and sometimes mortality [6]. Their prevalence is up to 20% among TSC patients [10], and their onset and growth are more common in the first decades of life [11]. Despite being benign, SEGAs can grow and, due to their proximity to the foramen of Monro, can obstruct cerebrospinal fluid flow, increasing intracranial pressure, exacerbating seizures, and leading to cognitive and behavioral decline [12]. Epilepsy is one of the most common neurologic symptoms in patients with TSC, with the reported incidence between 62% and 93% [5,13]. Epilepsy usually begins during the first months of life and, in most patients with TSC, before the first year, and is a significant cause of morbidity and mortality [9,14]. Although the current surgical and pharmacological management of seizures in TSC often provide significant benefits, about two-thirds of patients develop drug-resistant epilepsy, associated with increased rates of intellectual disability and other tuberous sclerosis-associated neuropsychiatric disorders (TAND), including autism and attention-deficit/hyperactivity disorder [13]. The presence of cortical tubers, SENs, and SEGAs are mainly responsible for these neurologic manifestations [3].

Renal disease, including renal angiomyolipomas (AMLs), is another leading cause of mortality in TSC patients [15]. Cardiac rabdomyomas, often asymptomatic, are also frequent, especially in children with TSC [1]. Among other manifestations, patients with TSC may present with lung cysts and lymphangioleiomyomatosis (LAM) in the lungs. LAM is a slowly progressive, low-grade, metastasizing neoplasm mostly occurring in women of about 35 years of age, with an incidence of about 30% in patients with TSC [16].

**Figure 1:**
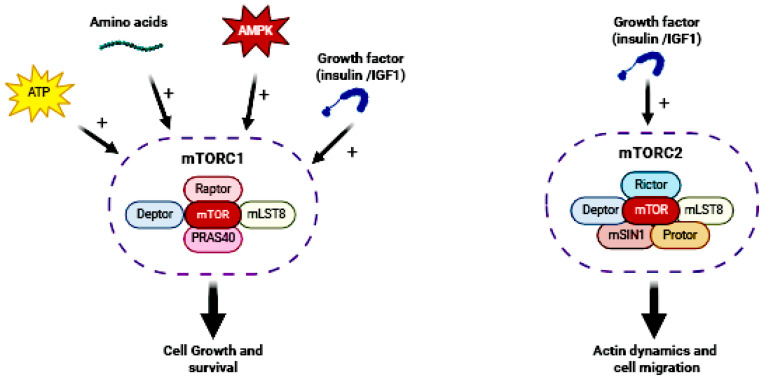
mTOR pathway.

The mTOR pathway is an evolutionarily conserved intracellular signaling pathway that is regulated upstream by phosphatidylinositol 3-kinase (PI3K)–Akt signaling [3]. The serine/threonine kinase mTOR exerts its functions through two distinct complexes [17,18], mTOR complex 1 and 2 (mTORC1 and 2, respectively; Figure 1); both are composed of mTOR acting as the catalytic core of the complex, but differ in their associated proteins and sensitivity to the inhibitor rapamycin. mTORC1 consists of two core components, regulatory-associated protein of mTOR (Raptor) and mammalian lethal with SEC13 protein 8 (mLST8), and two inhibitory subunits, Akt/PKB substrate 40 kDa (PRAS40) and DEP domain-containing mTOR-interacting protein (Deptor). mTORC1 is a rapamycin-sensitive complex involved in the regulation of protein synthesis, lipid synthesis, autophagy, energy metabolism, and lysosome biogenesis, and receives inputs from upstream regulatory proteins that are influenced by growth factors (for example, insulin, insulin-like growth factor 1 and 2 (IGF1 and IGF2)), ATP concentrations, nutrients, and AMP-activated protein kinase (AMPK). When activated, mTORC1 promotes cell growth and survival via the regulation of mRNA translation, nucleotide biosynthesis, and autophagy. In the brain, mTORC1 has important functions related to synaptic transmission and plasticity, neural network activity, and neurogenesis. mTORC1 localizes to the surface of lysosomes and to the endoplasmic reticulum (ER) and Golgi apparatus (GA). mTORC2 shares mLST8 and Deptor with mTORC1, but has three unique elements: the Raptor-independent companion of mTOR (Rictor), the mammalian stress-activated protein kinase-interacting protein 1 (mSIN1), and the protein observed with Rictor (Protor). mTORC2 is a rapamycin-insensitive complex largely known for its role in regulating actin dynamics and cell migration [2], and it is found in the plasma membrane, mitochondria, and in a subset of endosomal vesicles [19].

Both TSC1 and TSC2 inhibit the mTORC1 pathway under physiological conditions. Thus, loss-of-function pathogenic variants in TSC1 and TSC2 cause mTOR hyperactivation, leading to TSC [20].

## 2. Available Therapeutic Options

A multidisciplinary approach with regular follow-up from childhood to adulthood is mandatory for the management of TSC [4]. Several therapeutic options are available for the treatment of focal seizures and infantile spasms associated with TSC, including vigabatrin, hormonal therapy, epilepsy surgery, a ketogenic diet, and vagus nerve stimulation [9,21]. However, about two-thirds of patients develop refractory epilepsies, associated with increased rates of intellectual disability and other TANDs [13]. In recent years, mTOR inhibitors (mTORi), such as sirolimus and everolimus, have also been increasingly used for the treatment of the other various manifestations of TSC, including kidney AMLs, SEGAs, facial angiofibromas, and lymphangioleiomas [15,16]. For some of these lesions, when necessary, nonpharmacological interventions, such as surgery and selective arterial embolization (for renal AMLs), are also available [4] (Table 1).

### 2.1. Vigabatrin

According to the International TSC Consensus Guidelines, the first-line treatment for TSC-associated epilepsy is vigabatrin, which is more effective if introduced early [4]. Different mechanisms may account for vigabatrin’s effects on TSC. It is an irreversible inhibitor of Gamma-Aminobutyric Acid (GABA)-transaminase, an enzyme that degrades GABA, and a promoter of the synaptic release of GABA, finally resulting in elevated brain GABA levels. The reduction in glutamate/glutamine cycling between astrocytes and neurons induced by vigabatrin also contributes to the antiseizure effects associated with this drug [22]. Vigabatrin also inhibits the mTOR pathway activity, which could further account for the efficacy of vigabatrin in TSC. The recently completed EPISTOP (Long-Term, Prospective Study Evaluating Clinical and Molecular Biomarkers of Epileptogenesis in a Genetic Model of Epilepsy–Tuberous Sclerosis Complex) study found that preventive vigabatrin treatment resulted in a reduced risk of seizures, infantile spasms, and drug-resistant epilepsy; however, there was no difference in the prevalence of developmental delay or autism at the age of 2 years [23]. In addition, the PREVeNT study (the first multicenter phase IIb study on the preventive administration of vigabatrin to infants with TSC) compared vigabatrin treatment at the time of the initial detection of seizure activity with an electroencephalogram versus seizure onset. The aim was to assess whether earlier intervention could improve the developmental outcomes of and decrease the likelihood of severe seizures in infants with TSC. This clinical study found that early treatment with vigabatrin delayed the onset and reduced the occurrence of infantile spasms in infants with TSC. However, this preventive effect was not observed for focal seizures or drug-resistant epilepsy, and the prevention of infantile spasms did not result in measurable improvements in cognitive outcomes at the age of 2 years [24]. Therefore, additional evidence is needed before preventive treatment with vigabatrin can be recommended for all infants with TSC.

Among vigabatrin’s side effects, potential retinal toxicity associated with peripheral vision loss deserves particular attention. While the risk of retinal toxicity or abnormalities on a brain MRI may correlate with the total cumulative dose, the improved control of infantile spasms also correlates with the dose [23]. Actually, even though vigabatrin is the recommended first-line therapy, studies in Europe have suggested that it is not always the most commonly prescribed antiseizure medication (ASM) in patients with TSC, probably due to the risk of visual field defects, which may not be reversible upon discontinuation [25].

### 2.2. mTOR Inhibitors: Everolimus and Sirolimus

Several studies have confirmed that mTORC inhibition could be a valid therapeutic strategy in TSC. Rapamycin (sirolimus) inhibits mTORC1 activity by forming a complex with the FK506 binding protein 1A 12 kDa (FKBP12). The FKBP12–rapamycin binding complex interacts with mTOR and inhibits mTORC1 by an allosteric mechanism [9]. Everolimus, a synthetic analogue of rapamycin, is an mTOR inhibitor (mTORi) approved by the EMA as Votubia (EMA/229443/2018) as an adjunctive treatment in patients from 2 years of age with partial-onset seizures related to TSC that have not responded to other treatments, and for the treatment of SEGAs and AMLs. Similarly, based on the results obtained in the EXIST-3 (NCT01713946) clinical trial, everolimus was approved by the FDA for the adjunctive treatment of adult and paediatric patients aged 2 years and older with TSC-associated partial-onset seizures, in addition to its approval for SEGAs and AMLs [26]. In the first large-scale precision medicine trial on a genetically mediated epilepsy, everolimus was effective at reducing seizure frequency in people with TSC [1]. While everolimus can be a therapeutic option for therapy-resistant epilepsy in TSC patients, it can take a long time for seizure freedom to occur [27].

As mentioned above, TSC causes tumours to grow at multiple sites, resulting in variations in the severity of the condition among patients. Both in humans and in animal models, mTORi, specifically sirolimus and everolimus, can also reduce TSC-related lesions, such as kidney AMLs, SEGAs, and facial angiofibromas [28]. Double-blind, placebo-controlled clinical trials have demonstrated the effectiveness of mTORi in treating TSC-related brain and kidney tumours, such as SEGAs and AMLs, that are not candidates for surgery [29,30,31]. Regarding cardiac rhabdomyomas, mTORi are considered a temporary and safe treatment for symptomatic cardiac rhabdomyomas in children with TSC, especially for high-risk or inoperable tumours. However, high-quality randomized trials are needed to further validate these effects [32].

Considering the key role of increased mTORC1 signaling in LAM pathophysiology, the LAM mainstay treatment is represented by mTORi and, since 2015, sirolimus has been the treatment approved by the FDA at the onset of LAM immunological therapy. Several clinical studies have supported sirolimus’s efficacy in controlling pulmonary function and decreasing lymphatic symptoms in LAM patients [28,33,34,35].

mTOR inhibitors have significant side effects, including mouth ulceration and stomatitis, with an additional risk of immunosuppression and severe infection [1]. While the adverse effects of everolimus are known and well studied, this cannot be said for sirolimus. To date, however, the adverse effects associated with the use of sirolimus in infants and children with TSC are common but not life- or health-threatening (anemia, hyperlipidemia, and thrombocytosis), so sirolimus appears to be safe and well tolerated in young patients with TSC [36].

### 2.3. Cannabidiol

In April 2021, cannabidiol (CBD) was authorised in the EU as an adjunctive therapy for the treatment of seizures associated with TSC for patients of 2 years of age and older. Considering its poor affinity toward the CB1 receptor, one of the hypothesized mechanisms underlying the antiepileptic effect of CBD involves the antagonism of the G Protein-Coupled Receptor 55 (GPR55), which regulates glutamate release in a calcium-dependent way, modulating neuronal excitability. Physiologically, GPR55 is activated by some cannabinoids and lysophosphatidylinositol (LPI), an endogenous endocannabinoid neurotransmitter. LPI, among other functions, increases the excitatory–inhibitory ratio of the hippocampus through the increased mobilization of intracellular calcium. During acute seizures, GPR55 is more active and the LPI concentration is increased, forming a damaging positive feedback loop. CBD blocks the effects of LPI, reduces glutamate release, and restores the balance between excitation and inhibition, thereby dampening neuronal excitability and the occurrence of repeated seizures [37]. Additional hypothesized mechanisms underlying CBD’s antiepileptic effect involve the activation and rapid desensitization of the Transient Receptor Potential Vanilloid Type 1 (TRPV1) channels that have a role in the regulation of cortical excitability [38], and the inhibition of the adenosine reuptake pump Equilibrative Nucleoside Transporter 1 (ENT1). This latter mechanism would increase the extracellular adenosine concentration and enhance adenosine-mediated signaling through A1 and other centrally expressed adenosine receptors, thus contributing to seizures termination [39,40]. In addition to these effects, a modulation of the mTOR pathway has also been hypothesized [39,41].

Like everolimus, cannabidiol is effective and well tolerated in TSC patients [42]. However, no comparative effectiveness data exist to recommend a specific ASM, everolimus or cannabidiol, over one another in a particular patient. Furthermore, both everolimus and cannabidiol have important drug interactions with other ASMs. Cannabidiol is an inhibitor of CYP3A4 and CYP2C19 and drug–drug interactions occur mainly with clobazam and valproic acid [43]. A recent analysis of the Eudravigilance database suggested that the most common side effects reported for cannabidiol are hepatic disorders (due to increased blood levels of liver enzymes), somnolence, drug ineffectiveness, fever, decreased appetite, diarrhoea, and vomiting [43].

### 2.4. Adrenocorticotropic Hormone

To resolve the hypsarrhythmia pattern in the EEG (when present) and remit infantile spasms lasting over two weeks, adrenocorticotropic hormone (ACTH) or prednisolone can be added as a second-line therapy [4]. Although the exact mechanism associated with ACTH remains uncertain, many have been suggested, including the glucocorticoid-induced improvement of the blood–brain barrier, the downregulation of corticotropin-releasing hormones, and interference with the synthesis of neuroactive steroids [44,45]. ACTH is less effective than vigabatrin in TSC and has several adverse effects, such as hypertension, hyperglycemia, irritability, electrolyte imbalance, severe life-threatening or other infections due to immunosuppression, sleep disturbance, reversible hypertrophic cardiomyopathy, and cerebral cortical atrophy [46].

### 2.5. Ganaxolone

A phase III study (TRUSTTSC) of ganaxolone in patients with TSC is ongoing, which is a synthetic analog of allopregnanolone, a metabolite of progesterone. Ganaxolone acts as a positive allosteric modulator of Gamma-Aminobutyric Acid (GABA) A receptors in the CNS by binding at a different site from that of benzodiazepine. It is approved to treat epileptic seizures in children affected by cyclin-dependent kinase-like 5 (CDKL5) deficiency disorder [5].

### 2.6. Nonpharmacologic Treatments

Regarding patients with TSC-related epilepsy, the evaluation of nonpharmacologic options early in treatment is important due to the poor response to ASMs, including the resistance of infantile spasms to vigabatrin and hormonal therapies [4]. The nonpharmacologic epilepsy treatments shown to be effective in patients with TSC are the ketogenic diet, epilepsy surgery, and vagus nerve stimulation [21]. The ketogenic diet represents a widely used and effective treatment for intractable epilepsy, TSC, and other traumatic brain injuries, but its mechanisms of action are not well understood. Interestingly, several studies have shown that the anticonvulsant effect of the ketogenic diet may also be related to its ability to inhibit mTOR signalling in the brain [47] by modulating the insulin/AKT/mTORC1 pathway [48]. Vagus nerve stimulation for epilepsy can be used in TSC if surgery is unsuccessful or not an option. There are data suggesting that responsive neurostimulation may be effective in selected adults with TSC and intractable seizures [49]. For TSC-related lesions, surgery and selective arterial embolization (SAE) can be considered. For example, neurosurgery is recommended for enlarging SEGAs that are causing life-threatening neurologic symptoms [4]. Haemorrhage from AMLs can pose a life-threatening risk. Consequently, treating symptomatic patients or those with lesions larger than 4 cm has become a widely adopted practice. In recent years, SAE has gained favour as a treatment option for AMLs in both elective and emergency settings due to advancements in microcatheters and the improved image quality of diagnostic equipment [50]. SAE is particularly recommended for patients with TSC who have multiple renal AMLs and need a nephron-sparing approach. SAE offers the advantages of a minimally invasive, high-efficacy, low-complication surgery with a good long-term outcome.

## 3. Towards Development of New Drugs for TSC

While the molecular mechanisms by which mTOR hyperactivity causes abnormal cell growth can be supposed, the precise molecular mechanisms by which mTOR hyperactivity causes neuronal hyperexcitability, seizures, and brain and kidney lesions remain to be fully defined. Recently, several studies using TSC patients’ biological samples and preclinical studies based on TSC animal models have addressed this issue, and have offered insight as well into new potential treatments and targets.

### 3.1. Epilepsy and Brain Tumours: Involvement of Neuroinflammation, Ion Channels, and EGFR Pathways

Recent DNA- and RNA-sequencing studies [51,52,53,54] have emphasized the contribution of changes in the genes coding for ion channels and the proteins involved in neuroinflammation to TSC epileptogenesis and brain tumours, thus unveiling novel possible pathways to target. A brain transcriptome analysis of 22 TSC patients’ samples (aged 1–47 years old) demonstrated an upregulation of the genes associated with inflammatory and immune responses, including the complement system, in parallel with a downregulation of the genes associated with neurogenesis and glutamate receptor signalling. In addition, the miR-34 family of non-coding microRNA, which modulates neurite outgrowth in mouse primary hippocampal neuronal cultures, was significantly overexpressed [51]. A DNA methylation study using patients’ brain samples provided evidence of an enrichment of DNA methylation for the immune system, the MAPK pathway, and the extracellular matrix organization, suggesting the critical role of these pathways in SEGAs development [52]. In another study using human brain samples and RNA-sequencing analysis, significant neuroinflammation was been found in TSC-associated brain tumours [53]. Furthermore, the use of a TSC animal model (conditional TSC1 knock out) provided evidence that changes in the expression of multiple ion channels and cytoskeleton proteins may drive epileptogenesis in TSC. In fact, the deletion of *Tsc1* in mice resulted in changes in excitability and synaptic adaptation in the hippocampus, which emerged before seizure onset, progressed over time, and were rescued after early treatment with the mTOR inhibitor rapamycin [54]. Later in epileptogenesis, a hippocampal increase in the excitation-to-inhibition ratio has been observed. A transcriptome analysis of the genes encoding the ion channels or proteins related to the action potential and cytoskeleton showed that 27 genes were differentially expressed just before seizure onset, suggesting a potential driving role in TSC epileptogenesis [54]. In particular, the combined altered expression of sodium and potassium channel genes Ryr1 and Ryr3 (and many other channels), and of cytoskeleton proteins such as dystrophin and filamin A, were emphasized as having a role in seizure onset. Most of these changes were rescued upon rapamycin treatment [54]. Unlike the electrophysiological changes caused by severe channelopathies in rare monogenic epilepsy syndromes [55,56,57], the hyperexcitability phenotype present in this TSC mouse model may result from small cumulative changes, not in one but in several ion channels and other synaptic proteins [54]. Of note, altered ion channel expression in association with mTOR hyperactivation (Kv1.1 and Kv4.2) has also been found in a mouse model of cortical tubers and epilepsy (NS-Pten KO mice). In this case as well, the change was normalised upon rapamycin treatment [58,59,60,61]. Altered calcium influx via L-type Ca^2+^ channels and enhanced neuronal network activity has been shown in TSC2^−/−^ neurons. Again, the long-term treatment with rapamycin reversed the altered neuronal activity [62]. In another study, a reduction in dendritic L-type Ca^2+^ channels activity as a consequence of the increased expression of the RNA-binding protein (RBP) Parkinsonism-associated deglycase (DJ-1) modulatory protein has been observed [63]. These findings suggest that calcium channels can represent novel targets for the treatment of epilepsy in TSC and, perhaps, other mTORopathies. Beyond the immediate control of excitability, emerging evidence has shown that ion channels are differently expressed in the form of gliomas [64,65,66]. These results indicate that ion channels and regulators of the immune system may be further explored as candidate targets for drugs with beneficial effects on cell growth besides mTOR inhibition.

In addition, the epidermal growth factor receptor (EGFR) overexpression observed in the SEGAs and cortical tubers of TSC patients has opened the possibility of targeting the EGFR signalling pathway with afatinib. Afatinib belongs to a new class of targeted anticancer drugs, the so-called tumour growth inhibitors. This class of molecules acts against tumours more selectively than traditional chemotherapy, as it recognizes specific proteins found in the membrane of tumour cells or inside the cells, i.e., it blocks the mechanisms by which cells reproduce [67]. Since these proteins are only found in small amounts in healthy cells, it appears that the action is targeted towards tumour cells. Afatinib irreversibly blocks signalling from all ErbB family members, EGFR (ErbB1), HER2 (ErbB2), ErbB3, and ErbB4. In a cell-based assay using primary TSC patient-derived cells, afatinib reduced their proliferation and viability in primary SEGAs and tuber cells, suggesting afatinib could be a promising therapeutic alternative to everolimus for TSC [68]. Interestingly, several in vivo/in vitro pharmacologic experiments and molecular docking simulations suggested that CBD could also inhibit the EGFR/AKT/MMPs signaling pathway, triggering programmed cell death, inhibiting angiogenesis, and reducing tumour growth and metastasis in different mouse models and tumour cell types [69,70,71,72]. This evidence provides a novel molecular mechanism responsible for the beneficial effects of CBD in TSC-related epilepsy and corroborates its therapeutic potential in this disease.

### 3.2. Potential Role of LKB1 in TSC: Orchestrating mTOR Pathway Dynamics

As aforementioned, AMPK plays a key role in the activation of TSC1 and TSC2; in this context, investigating the upstream regulatory aspects of the kinase may be relevant to unveiling the underlying mechanisms of the pathology, and to identifying potential new therapeutic targets. Currently, the main activator of AMPK is the liver kinase B1 (LKB1), a serine–threonine kinase highly conserved across different species. In addition to AMPK, LKB1 activates another 13 kinases within the AMPK family, each with a plethora of cellular functions [73]. Consequently, LKB1 plays a crucial role in various processes, such as adhesion, the regulation of energy metabolism, and apoptosis. LKB1 is expressed in several metabolic tissues, such as adipose tissue and skeletal myofibers, where it acts as a crucial hub for the interplay between cell metabolism and function. Recently, it was demonstrated, in fact, that the downregulation of the kinase was potentially involved in the aberrant mechano-metabolic coupling that characterizes dystrophic myofibers [74]. Under energetic stress conditions, LKB1 negatively regulates mTOR signalling [75] via AMPK phosphorylation and activation. In addition, two well-characterized targets of the mTOR-TSC2 axis, S6 kinase (S6K) and 4E-BP1 (eukaryotic translation initiation factor 4E-binding protein 1), key translational regulators involved in cell proliferation, were inhibited when LKB1 was overexpressed [76].

LKB1 is deregulated in certain specific forms of lung cancer and in Peutz–Jeghers syndrome, an inherited genetic disorder characterized by the development of benign polyps in the gastrointestinal tract. The histological similarities between these latter polyps and the hamartomatous polyps of TSC suggest that a potential connection between the two diseases might lie in the common deregulation of LKB1, leading to abnormal cell growth [76] and proliferation. Nonetheless, the low levels of LKB1 would make the kinase a hidden target, paving the way for further analyses to investigate the potential upstream regulatory mechanisms, post-translational modifications, or epigenetic modulation (e.g., miRNAs). Currently, targeting the downstream players of LKB1 seems to be the most feasible approach by which to propose an effective therapeutic strategy for TSC, for example, using AMPK activators such as metformin.

### 3.3. Metformin: Beyond AMPK Modulation

Clinical and preclinical studies support metformin repurposing in TSC [77]. Metformin is a widely prescribed anti-hyperglycemic drug that activates AMPK via the mitochondrial respiratory chain and inactivates mTORC1, thus protecting from abnormal cell proliferation and differentiation. Recently, the first multicentre randomized, double-blind, placebo-controlled trial was performed to assess the safety and efficacy of metformin in 51 TSC patients aged over 10 years (children and adults) for 12 months [77]. Metformin reduced SEGAs volume (which was more marked in younger, i.e., <30 years of age, patients) and seizure frequency compared with the placebo. Even though some studies have revealed that metformin exerts a beneficial effect in diverse kidney diseases [78], metformin did not reduce renal angiomyolipomas volume in this clinical study [77]. Also, controversial results have been obtained when testing metformin efficacy against kidney failure in animal models [79,80].

**Figure 2:**
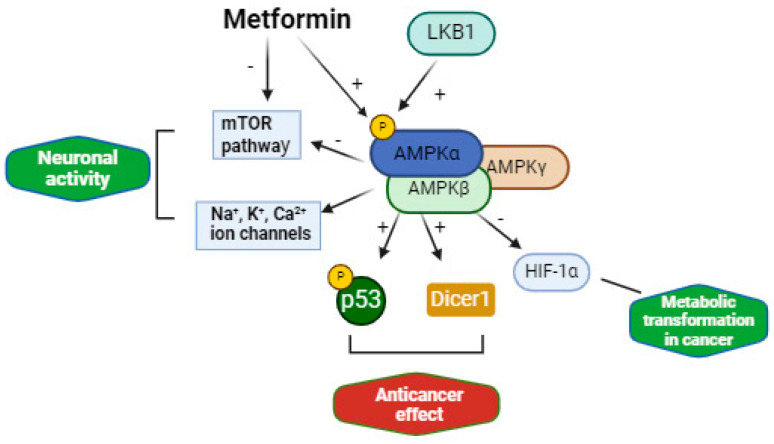
Mechanisms of action of metformin and its potential role in TSC.

The different mechanisms of metformin can provide reasons for repurposing metformin in TSC to restore abnormal cell proliferation and differentiation and to treat epilepsy [81]. Metformin can inhibit mTORC1 via the AMPK pathway and independently of AMPK. Metformin has an antineoplastic effect by targeting other genes involved in many cancers, such as p53, DICER1, and c-MYC, which play a crucial role in growth control, differentiation, and apoptosis. Metformin has shown beneficial effects in models of pancreatic cancer, renal cell carcinoma, myeloma, breast cancers, and bile duct cancer, among others [82]. Metformin is also an inhibitor of the oxygen-sensitive transcriptional activator HIF-1α (hypoxia inducible factor) via AMPK and mTORC1, which has a key role in metabolic transformation in cancer. Metformin also inhibits the expression of fatty acid synthase, a multi-enzyme protein that catalyses fatty acid synthesis and is upregulated in cancer cells. Metformin reduces the production of ROS (reactive oxygen species), oxidative stress, and DNA damage through the inhibition of mitochondrial complex I, and fibrosis and inflammation through augmented AMPK phosphorylation [81,83]. Metformin can also have an antiepileptic effect that can be mediated by the inhibition of mTOR, the activation of AMPK and energy pathways, the prevention of oxidative damage induced by seizure activity, and the regulation of ion channels via AMPK [84]. In particular, AMPK has been shown to selectively modulate a variety of potassium (e.g., Kv2.1), calcium (L-type voltage-gated calcium channels, TRPC4 and CRAC), and sodium (Nav1.7) channels (Figure 2), which are crucial for maintaining excitability balance, synaptic plasticity, and neurotransmitter release. Metformin has also been shown to suppress seizures in some rodent models of epilepsy [85,86] and in a zebrafish PTZ-induced seizure model [87]. Metformin was granted an orphan drug designation for the treatment of progressive myoclonic epilepsy type 2 (Lafora disease) in 2016 [88].

Metformin effectively crosses the blood–brain barrier and is distributed in multiple brain regions after oral dosing [89]. Even though this drug appears to be a less potent inhibitor of mTOR compared with everolimus and rapamycin, it has a more favourable benefit–risk profile and is less expensive. Metformin has the advantage of not interacting with the cytochrome P450 system and, therefore, it is unlikely to interfere with the metabolism of other mTOR inhibitors and antiseizure drugs. For the same reason, and in contrast to the mTOR inhibitors everolimus and rapamycin, the metabolism of metformin is not disturbed by antiseizure drugs, such as cannabidiol or carbamazepine, that many TSC patients may be taking [41,43].

### 3.4. Renal Cysts: Involvement of the ClC-5 Transporter

Together with central nervous system tumours, renal AMLs are one of the most prevalent hamartomas in TSC; most TSC patients also develop polycystic kidney disease. Both diseases are associated with significant morbidity and mortality, but there is still no effective treatment for aberrant kidney growth [15]. Cyst formation is a complex mechanism that is accompanied by altered tubulogenesis, increased apoptosis, and cellular proliferation. An elegant study recently disclosed the involvement of TFEB, a transcriptional modulator of lysosomal biogenesis and autophagy, in the mechanisms of mTORC1 hyperactivation in kidney disease [90]. It is well known that the crucial step in the formation of kidney cysts entails the secretion of fluid into the cyst lumen, which is dependent on chloride secretion from the cyst epithelium into the cyst lumen. In autosomal dominant polycystic kidney disease (ADPKD), the main mechanism mediating Cl^−^ secretion into cysts is via the cystic fibrosis transmembrane conductance regulator (CFTR) in principal cells [91]. Accordingly, a significant reduction in cysts expansion was demonstrated by using CFTR inhibitors [92], whose modulation of channel gating was also elucidated [93]. Importantly, recent studies have shed light on a selective mechanism underlying cystic expansion involving ClC-5 in both humans and mouse models of TSC. ClC-5 is a 2Cl^−^/H^+^ exchanger located in the endosomal membrane in the proximal tubule and in α-intercalated cells of the collecting duct, where it plays a critical role in dissipating the membrane depolarization generated by H^+^-ATPase [94,95]. ClC-5 and H^+^-ATPase may function synergistically in cyst epithelia by secreting Cl^−^ and H^+^ into the cyst lumen. Particularly, it was demonstrated that the initiation phase of cystogenesis precedes the robust expression of Foxi1, the chief regulator of acid–base transporters in intercalated cells, along with a parallel increase in ClC-5 apical membrane expression in cystic epithelium [96]. The difference in the mechanism of Cl^−^ secretion into the kidney cyst lumen with respect to ADPKD reflects the distinct cell types lining the cysts, which in TSC are overwhelmingly composed of α-intercalated cells [96,97,98]. Cyst expansion is strictly related to Cl^−^ secretion mediated by ClC-5, because an increasing salt concentration in the cyst lumen osmotically drives water influx, and thereby leads to a volume increase [96]. Thus, the presence of the apical electrogenic chloride exchanger ClC-5 in the cells lining the cysts has brought attention to the role of this protein in TSC etiopathogenesis, finally recognizing ClC-5 as a new player in renal cyst formation [96,99]. The inhibition of ClC-5 may have therapeutic effects by decreasing the cyst burden in TSC. In contrast to the relative extensive pharmacological characterization performed for other renal ClC proteins, such as ClC-K chloride channels [100,101,102], no ClC-5 high-affinity ligand is currently available; thus, the identification of drugs that specifically inhibit ClC-5 to slow down or to prevent cyst expansion is an unmet need.

## 4. Conclusions

Several therapeutic options are available for the treatment of epilepsy and hamartomas associated with TSC. Though, there is still a need for novel therapeutic targets and approaches. In this context, an improved understanding of the molecular mechanisms underlying epileptogenesis, brain tumours, and kidney cysts formation has shed light on ion channels and other synaptic proteins, AMPK, inflammatory markers, and EGFR as potential drug targets to restore hyperexcitability and cell growth, thus providing new avenues of drug discovery for TSC.

## Figures and Tables

**Table 1:** Available therapeutic options for TSC.

Drug	Therapeutic Approach	Indication
Vigabatrin	symptomatic	First-line monotherapy to treat TSC-related spasms or focal seizures in children under the age of one year.
Cannabidiol	symptomatic	Adjunctive treatment of TSC-associated seizures in children more than 2 years of age.
ACTH	symptomatic	Second-line therapy to remit long-lasting infantile spasms.
Ketogenic diet	symptomatic	Reduction in seizure frequency, but poor long-term efficacy data.
Surgery	symptomatic	Removal of epileptogenic foci, SEGAs, and cardiac tumours after careful presurgical assessment.
Selective arterial embolization	symptomatic	Safe treatment option for patients with symptomatic or large AMLs.
Everolimus	disease modifying	Reduction in the growth of typical TSC lesions and adjunctive treatment for children and adults with drug-resistant epilepsy.
Sirolimus	disease modifying	Improvement of TSC-related lymphangioleiomas and stabilization of lung function.

SEGA, subependymal giant cell astrocytoma; AML, angiomyolipomas.

## Data Availability

Not applicable.
